# Food habits in overweight and obese adolescent girls with Polycystic ovary syndrome (PCOS): a qualitative study in Iran

**DOI:** 10.1186/s12887-020-02173-y

**Published:** 2020-06-04

**Authors:** Leila Hajivandi, Mahnaz Noroozi, Firoozeh Mostafavi, Maryam Ekramzadeh

**Affiliations:** 1 Student Research Committee, School of Nursing and Midwifery, Isfahan University of Medical Sciences, Isfahan, Iran; 2 Department of Nursing and Midwifery, Kazerun Medical Sciences Branch, Islamic Azad University, Kazerun, Iran; 3 Department of Midwifery and Reproductive Health, School of Nursing and Midwifery, Isfahan University of Medical Sciences, Isfahan, Iran; 4 Department of Health Education and Promotion, School of Health, Isfahan University of Medical Sciences, Isfahan, Iran; 5 Nutrition Research Center, Department of Clinical Nutrition, School of Nutrition and Food Sciences, Shiraz University of Medical Sciences, Shiraz, Iran

**Keywords:** Polycystic ovary syndrome, Food habits, Adolescent, Qualitative research

## Abstract

**Background:**

Polycystic ovary syndrome (PCOS) is a hormonal disorder in women of reproductive age. It seems that over the recent years, PCOS has augmented in adolescent girls due to unhealthy food habits and obesity. So, the present study was conducted to explore the food habits in overweight and obese adolescent girls with PCOS.

**Methods:**

In the present qualitative study, 33 participants were selected using a purposive sampling method. Data were collected through individual in-depth interviews, focus group discussions (FGDs), and field notes. These data were analyzed through the use of conventional qualitative content analysis.

**Results:**

Three main categories were extracted: First, the high consumption of unhealthy food had three sub-categories: “high consumption of fatty and salty foods”, “high consumption of unhealthy snacks”, and “high consumption of sugar-rich foods”. Second, low consumption of healthy food had three sub-categories: “low consumption of dairy products”, “low consumption of fiber-rich foods”, and “low consumption of meat, beans, fish and seafood” Third, inappropriate behavioral habits had three sub-categories: “lack of concentration and consumption of large meals”, “inappropriate dietary and physical activity patterns”, and “skipping the meals and going on arbitrary diets”.

**Conclusion:**

This research through presenting an image of food habits in overweight and obese adolescent girls with PCOS is able to help for designing the necessary interventions to change the food habits, control the symptoms and complications of PCOS, and finally, improve the reproductive health of these girls.

## Background

Polycystic ovary syndrome (PCOS) is a hormonal disorder in women of reproductive age [1]. Based on the diagnostic criteria, the prevalence of PCOS among adolescent girls (11–19 years old) is 1.8–15% [2]. In women and adolescent girls with PCOS, obesity is a pathophysiologic principle, hence the fact that obesity leads to an increase in insulin secretion, resulting in the production of ovarian androgens. Accordingly, a permanent imbalance occurs in sex hormones, causing a disorder in the ovulation process which ultimately entails infertility [3, 4].

Obesity is one of the main problems of industrial life. The increasing rates of obesity are largely attributed to lifestyles; including unhealthy dietary habits, like consuming the widely distributed junk, fast food coupled with increasing sedentary lifestyles [5]. Inappropriate food patterns and behaviors, which result in obesity, are formed in adolescence and continue into adulthood [6, 7]. It seems that over the recent years, PCOS has augmented in adolescent girls due to unhealthy food habits and obesity [8, 9]. Overweight and obesity are common among many groups of young women with PCOS and may precipitate subsequent reproductive and metabolic disorders that require expensive and invasive care. Attention to nutrition and diet is important in management of these young women [10]. Eleftheriadou et al. indicated adolescent girls with PCOS more likely to eat an evening meal and eat this over an hour later when compared to controls. This study showed that PCOS adolescent girls in general have worse dietary habits than controls [11]. Since overweight and obesity play important roles in the occurrence, progress, and PCOS-induced complications, having ideal weight and healthy nutritional practices can reduce the PCOS-induced complications in adolescent girls. Several studies have introduced weight loss and lifestyle modification as the first step to PCOS treatment [5, 12–15]. Considering the different aspects of lifestyle in adolescent girls with PCOS, determining the food habits of these girls could provide an appropriate context for systematic, comprehensive, cultural-based interventions and caring programs in the society. Because qualitative research is an approach to discovering and describing the experiences of participants and conceptualizing them, it could increase insight and awareness about human experiences. This approach is usually used when there is a need for explaining concepts and their relationships [16]. Thus, the present study was conducted to explore the food habits in overweight and obese adolescent girls with PCOS.

## Methods

This research was conducted using a qualitative approach.

### Settings, sampling, and recruitment

In the present study, adolescent girls with PCOS residing in Shiraz, Iran were selected through purposive sampling. The sampling continued considering the maximum variety in age, educational level, occupation, marital status, body mass index, and duration of the disease. Inclusion criteria were being adolescent girls with PCOS diagnosed by a gynecologist through diagnostic criteria [17], aged 15 to 21 (for diagnosis of PCOS must be at least 2 years past the menarche [17]), willingness to participate in the study, informed consent of the adolescents and their parents (in cases of ages below 18 years), adolescent girls with PCOS being overweight or obese, no history of major psychiatric disorders under medical treatment, no history of chronic diseases such as cardiovascular diseases, diabetes, kidney diseases, etc. prescribed with specific diets. An exclusion criterion was unwillingness to continue collaborating and participating in the study. In the present study, regarding relationships and interactions between adolescent girls with PCOS and healthcare providers (midwives, gynecologists, nutritionists, and endocrinologists) they were selected as information-rich participants through purposive sampling. In this regard, healthcare providers with different working experience recruited for the study. Participants were recruited directly or telephone numbers were obtained and they were subsequently telephoned. Accesses to adolescent girls with PCOS were granted through gynecology clinics and gynecologists’ offices. Healthcare providers were accessed through midwives, gynecologists, nutritionists and forensic medicine specialists’ offices.

### Data collection

Data were collected through individual in-depth interviews, focus group discussions (FGDs), and taking field notes between November 2016 and October 2017. After reaching eligible participants, none of them refused to participate in the study. The first author (LH) conducted the interviews and FGDs. Other authors have previous interviewing experience and qualitative paper/report writing. Prior to data collection, the first author wrote down initial preconceptions about the study topic based on her previous working experience and from literature review. The interviews with adolescent girls with PCOS started with a general question, “What do you eat during a day? Please explain”, after which, the open and interpretative responses of participant guided the process. The interviews with healthcare providers started with a general question, “Based on your experiences what are the food habits of adolescent girls with PCOS? Please explain”, after which, the open and interpretative responses of participant guided the process. Individual interviews (lasted 30 to 85 min) were performed in places and time desired by the participants. In the present study, amongst adolescent girls with PCOS and healthcare providers, those who were more willing to participate in group discussion than individual interviews participated in group discussions. FGDs were conducted in an agreed place such as gynecology clinics. At the group discussion sessions, the researcher was acting as the facilitator and guide of the discussions, and another person was present to take notes. In the present study, sampling continued until the researcher felt that there wasn’t any new data during the analysis, so that after 21 individual interviews (with 10 adolescent girls with PCOS and 11 healthcare providers) and two focused group discussions (one for eight adolescent girls with PCOS and one for four healthcare providers) data saturation was obtained and sampling was completed. In this study, all of the nonverbal behaviors of the interviewees were considered and field notes were taken in this regard. The demographic characteristics of 33 participants are shown in Tables 1 and 2.

**Table 1:** Demographic characteristics of overweight and obese adolescent girls with PCOS

Characteristic		Number
**Age (years)**	15–21	18
**Mean (SD)**	17.66 (±2.4)	
**Education status**	High school or below	13
	Student in college	5
**Occupation**	Student	15
	Unemployed	3
**Marital status**	Single	13
	Married	5
**BMI (kg/m**^**2**^**)**	More or equal than 25	11
	More or equal than 30	7
**Duration of the disease (years)**	Less than 1	4
	More than1	14

**Table 2:** Demographic characteristics of healthcare providers

Characteristic		Number
**Age (years)**	Equal or less than 50	11
	More than 50	4
**Occupation**	Midwife	2
	Gynecologist	6
	Nutritionist	5
	Endocrinologist	2
**Work experience (years)**	Equal or less than 10	3
	More than 10	12

### Data analysis

Data analysis was conducted using qualitative content analysis [16]. The interviews were transcribed verbatim by the first author (LH). Then, the narratives were frequently reviewed to present a general understanding of them. Afterward, sentences and phrases were coded, and similar codes were merged via an inductive approach. In this respect, codes with similar meanings were categorized in one group and sub-categories were created. With a comparison between sub-categories, groups with similar concepts were put in one category to create a main category.

### Rigor and trustworthiness

The findings were validated via different methods such as in-depth interviews in different times and places and a combination of data collection methods such as FGDs and field noting. In this study, transcripts were returned to participants for comments or corrections. Also, to approve the accuracy of the collected data, at different sessions, coded interviews were shared with four of the participants and their final opinions were achieved; so that member checking could be obtained. The opinions of two experts were also obtained to assure the consistency of the results. In the present study, to increase transferability of the data, results of the study were given to three individuals (adolescent girls with PCOS) with similar characteristics to the participants who did not participate in the study to judge the similarity of the results to their own experiences.

### Ethical considerations

The research was verified by the Ethics Committee Vice-Chancellor of Research at Isfahan University of Medical Sciences (Ethic Code: IR.MUI.Rec.1395.8.885). The reasons for the study were explained prior to each individual interview and FGD. Also, the issues of informed consent, confidentiality of information, anonymity, and right of avoidance from participation were practiced.

## Results

Following data analysis, 398 codes, nine sub-categories, and three main categories including “high consumption of unhealthy foods”, “low consumption of healthy foods”, and “inappropriate behavioral habits” were obtained (Table 3).

**Table 3:** Examples of codes, sub- categories, and main categories

Code	Sub-category	Main category
-Abundant use of sauce -Abundant use of fast food -Abundant use of prepared food -Abundant use of fried food	High consumption of fatty and salty foods	High consumption of unhealthy foods
-Eating sweets and ice-cream after meals -Eating cookies, chocolate, and sweet desert daily -Daily consumption of cake as a snack or breakfast - High consumption of soft drink and industrial fruit juice	High consumption of sugar-rich foods	
-Eating snacks immediately after a meal - Frequent use of junk food as a snack -Consuming cheese puffs and chips	High consumption of unhealthy snacks	
Non-consumption of milk -Replacing soft-drink with yogurt	Low consumption of dairy products	Low consumption of healthy foods
Low consumption of lamb, calf and cow meat -Low consumption of peas, beans, and lentils -Low consumption of fish and shrimp	Low consumption of meat, beans, fish and seafood	
-No fruit consumption in spite of their availability -Unwillingness to consume vegetable -No vegetable consumption in spite of their availability	Low consumption of fiber-rich foods	
- Speaking on the cell phone while eating - Eating while watching TV - Playing with the cellphone while eating -Craving for more food - Eating quickly	Lack of concentration and consumption of large meals	Inappropriate behavioral habits
-Sleeping right after eating -Long-lasting sitting -Limited physical activity	Inappropriate dietary and physical activity patterns	
-Eliminating breakfast from the daily food program -Arbitrarily food limitation -Eliminating dinner for losing weight	Skipping the meals and going on arbitrary diets	

### High consumption of unhealthy foods

The participants stated that, compared with traditional foods, adolescents were more interested in eating foods such as pizza, lasagna, fries, fast food, and prepared meals such as sandwiches, sausages, as well as sweets, candy, and ice cream. Leaning towards such kinds of food is due to their favorable taste, color, shape, easy accessibility, and advertisements. Participants further highlighted the role of friends and media in the consumption of these foods. High consumption of unhealthy foods has three sub-categories including “high consumption of fatty and salty foods”, “high consumption of unhealthy snacks”, and “high consumption of sugar-rich foods”.

### High consumption of fatty and salty foods

Most of the adolescent girls with PCOS stated that they eat fast food at least twice a week. Also, they usually prefer fast food for dinner. Participating girls mentioned the effective role of friends and peers in fast food consumption. They considered eating fast food with their friends and peers as enjoyable.

*“Eating fast food with my friends is fun. Whenever we go out, we eat fast food.” (19-year old girl).*

### High consumption of unhealthy snacks

Adolescent girls with PCOS stated that after a meal, they eat chips or puffs daily, which is very enjoyable for them. They considered the snacks very delicious. Healthcare providers narrated that advertisement in mass media (such as TV), easy access to these foods (even at school), and low price to be an effective factors in high consumption of snacks by adolescents.

“*Adolescents really like junk food. They can eat them every day, because food taste is very important for them. And, due to the high fat and salt content of junk food, they are really tasty.” (Endocrinologist).*

### High consumption of sugar-rich foods

Adolescent girls with PCOS expressed a great desire to eat sweets, cakes, biscuits and desserts. They reported eating cakes or biscuits and industrial fruit juice for breakfast. According to most of the participating girls, after a meal, they eat at least one pastry or ice cream daily. All participating girls stated that they consume soft drinks with fast food.

“*… After lunch, I usually eat ice-cream. I like eating chocolate and sweets. Whenever I feel hungry, I only think of sweet foods like cookies and cakes.” (19-year old girl).*

Healthcare providers also stated that adolescent girls with PCOS have a strong desire to eat simple carbohydrates, such as sweets and chocolate, especially at main meals and snacks, and generally tend to consume high amounts of sugary foods.

“*Adolescent girls with PCOS have a false appetite and a strong desire to eat sweets.” (Endocrinologist).*

### Low consumption of healthy foods

Adolescent girls with PCSO did not have a positive attitude toward consuming foods such as dairy products, meat, fruits, vegetables, legumes, and seafood. They stated that they know their importance in daily diet, but could not eat them due to their bad taste and smell. This main category has three sub-categories including “low consumption of dairy products”, “low consumption of fiber-rich foods”, and “low consumption of meat, beans, fish and seafood”.

### Low consumption of dairy products

Adolescent girls with PCSO stated that they have a very low tendency to drink milk and other dairy products due to their unpleasant smell and taste, and the ensuing gastro-intestinal problems which occur sometimes.

*“I don’t like milk, but I sometimes eat yogurt. I can’t drink milk, because I don’t like the taste.” (20-year old girl).*

Healthcare providers also narrated that these girls are not interested in milk and other dairy products.

“ … *Unfortunately, girls at this age are not interested in dairy products at all!!! They only eat ice cream!!!”(Nutritionists).*

### Low consumption of fiber-rich foods

Most of the participating girls indicated that they do not prefer to eat vegetables and fruits due to their bad taste. They mentioned that they only eat fruit and vegetables by family force.

*“I only eat fruits and vegetables if my mom forces me!!!” (16-year old girl).*

### Low consumption of meat, beans, fish and seafood

Adolescent girls with PCSO stated that they were reluctant to consume beans and meat because of their unpleasant taste. Healthcare providers also narrated that although meat is the main source of iron, and it is very effective in the reproductive health of adolescent girls with PCSO, they are not interested in eating it.

*“Unfortunately, in many cases, adolescent girls with PCSO are reluctant to eat meat.” (Nutritionists).*

Adolescent girls with PCSO stated that they were reluctant to consume fish and seafood. They attributed the very unpleasant taste and smell of fish and seafood to the lack of consumption of these foods.

“*I hate fish and seafood as they smell very bad‼ When I see them on the table I can’t eat anything. I even can’t eat a small piece of fish!!!” (18-year old girl).*

### Inappropriate behavioral habits

Participants pointed to high-lighted habits such as sleeping immediately after a meal, lack of physical activity, lack of concentration during eating, lack of attention to the amount of food consumed per meal, and over eating. A number of overweight and obese adolescent girls with PCSO try arbitrary diets or often eliminate dinner to lose weight. The sub-categories of inappropriate behavioral habits are “lack of concentration and consumption of large meals”, “inappropriate dietary and physical activity patterns”, and “skipping the meals and going on arbitrary diets”.

### Lack of concentration and consumption of large meals

Adolescent girls with PCSO narrated that they play with their cell phones, talk or watch TV while eating. This causes them to ignore the amount of food they eat during the meals.

*“I often eat lunch in front of the TV. Because, I don’t concentrate on eating, I eat a lot!!!” (17-year old girl).*

### Inappropriate dietary and physical activity patterns

Most adolescent girls with PCSO said that they have inactive lives, and almost all of them narrated that they sleep after eating. Also, healthcare providers stated that most adolescent girls are very inactive and they consider exercising difficult and time-consuming.

*“ … Unfortunately, most adolescent girls are very inactive, and they are not even willing to walk. In addition, they consider studying as their first priority and consider exercising difficult and time-consuming.” (Midwife).*

### Skipping the meals and going on arbitrary diets

Healthcare providers stated that most adolescent girls eliminate their meals (especially breakfast) for reasons that are: they have no tendency to eat in the early hours of the day, they are late and have no time for it and they have stayed up late. They mentioned that in order to lose weight, overweight and obese adolescent girls with PCSO go on arbitrary diets such as eliminating dinner or removing certain ingredients from their diet.

*“Of course, the role of the family is very important. Even the lifestyle and sleeping habits are important!!! Families that sleep till noon, eliminate breakfast.” (Gynecologist).*

## Discussion

The present study was conducted to explore the food habits in overweight and obese adolescent girls with PCOS. The results showed that adolescent girls with PCOS consume a lot of unhealthy foods such as fast food, soft drink, sweets, and junk food. There are emerging global data that women with PCOS have different baseline dietary energy intakes compared with women without PCOS. These alterations in diet may exacerbate clinical symptoms and compound risk of chronic disease in patients [18]. Eleftheriadou et al. found that adolescent girls with PCOS had sugar-rich breakfasts and consume more daily calories than the control group [11]. Zhang et al. also showed that high intake of total energy and fat were found in PCOS women (aged 12–44 years) [19].

The results of the present study showed that adolescent girls with PCOS consume a small amount of healthy foods such as fiber-rich foods, meat, beans, fish, seafood, and dairy products. Eleftheriadou et al. also showed that girls with PCOS were less likely to have cereals for breakfast and as a result consumed less fiber than controls [11]. Hmedeh et al. concluded in their study, a properly managed diet combined with a balanced lifestyle addresses insulin resistance, cardiovascular health and metabolism, all of which target PCOS symptoms and alleviate them [20].

The results of the present study showed that most overweight and obese adolescent girls with PCOS have little physical activity, skip the meals (especially breakfast), eat fast and consume large meals. In this regard, the results of the study by Amirjani et al. showed that in comparison with the control group, lifestyle scores were lower in the PCOS group in the fields of physical activity [21]. Also, Eleftheriadou et al. showed that girls with PCOS engaged in physical activities less than controls. Even when they did, the frequency and intensity of exercise was less [22]. Zhang et al. concluded that women with PCOS (aged 12–44 years) should avoid long-term sedentary lifestyle habits and focus on adding to the duration of, or enhancing the intensity of physical activity [19]. Vatopoulou & Tziomalos showed that weight loss and lifestyle changes appear to be effective in obese adolescents with PCOS [23]. So, consultation with overweight and obese adolescent girls with PCOS about the importance of nutrition control, as well as appropriate physical activity, is essential. In this regard, Carolo et al. concluded in their study, overweight and obese adolescent girls with PCOS who lost weight changed their dietary habits by adopting hypocaloric diets and eating more meals per day, as per nutritional counseling [24].

The results of the present study showed that overweight and obese adolescent girls with PCOS are heavily influenced by their friends and peers in choosing fast food and snacks. Also, Kilanowski in a study in adolescents showed that friends encourage unhealthy food choices [25]. In this regard, Bruening et al. concluded in their study, dietitians and health professional may consider developing strategies to engage friends to promote adolescents’ healthy dietary behaviors [26]. Also, in the present study, taste, color, shape and ease of access to fast foods, low price of junk foods (such as chips and cheese puffs), as well as mass media advertisement are among the reasons for leaning towards such kinds of food. Fitzgerald et al. concluded in their study, to develop effective nutrition interventions, it is important to gather child and adolescent input regarding factors perceived as influencing their food choices [27]. Several studies have shown that among adolescents, taste, color and shape are important factors in the process of food selection [28, 29]. Due to complexity of nutritional behavior [30], modifying food habits of overweight and obese adolescent girls with PCOS is a comprehensive approach, in which all the mentioned points should be considered. Also, future research includes development of interventions to assist these adolescent girls with healthy-eating decision making.

Through presenting an image of food habits in overweight and obese adolescent girls with PCOS, for the first time, the present study is able to help for designing the necessary interventions to change the food habits and prevention of disease complications in these girls.

Although the findings of the present qualitative study can provide a deep understanding of food habits in overweight and obese adolescent girls with PCOS, the results may not be shared by normal weight adolescent girls with PCOS.

## Conclusion

Overweight and obese adolescent girls with PCOS have inappropriate food habits, which can lead to complications such as infertility. Hence the necessities of designing and performing the required interventions to change the food habits, control the symptoms and complications of PCOS, and finally, improve the reproductive health of these girls.

## Data Availability

The datasets generated and/or analyzed during the current research are not publicly available as individual privacy could be compromised but are available from the corresponding author on reasonable request.

## Abbreviations

PCOS: Polycystic Ovary Syndrome
FGDs: Focus Group Discussions
